# Supplementary material for: Thermoelectric device for active thermal concealment, deception and messaging
Source: Nat Commun. 2025 Nov 10;16:9888. doi: 10.1038/s41467-025-66134-8 (PMC12603023; doi:10.1038/s41467-025-66134-8)
Supplement: Supplementary file 1 — Supplementary Information [file 41467_2025_66134_MOESM1_ESM.pdf]

Supplementary Information for

Thermoelectric Device for Active Thermal Concealment, Deception and Messaging

Yue Hou[1,2,#], Xiaosa Liang[1,#], Zhaoyu Li[3,#], Qianfeng Ding[1], Zheng Zhu[1], Xiaolong Sun[1], Chang Li[1], Wenjie Zhou[1], Wei Cao[1], Yuan Yu[4,*], Ziyu Wang[1,2,5,*]

[1]The Institute of Technological Sciences, Wuhan University, Wuhan 430072, China
[2]Key Laboratory of Artificial Micro-structures of Ministry of Education, School of Physics and Technology, Wuhan University, Wuhan 430072, China
[3]School of Power and Mechanical Engineering, Wuhan University, Wuhan 430072, China
[4]Institute of Physics (IA), RWTH Aachen University, 52074 Aachen, Germany
[5]School of Physics and Microelectronics, Key Laboratory of Materials Physics of Ministry of Education, Zhengzhou University, Zhengzhou 450001, China
[#]These authors contributed equally: Yue Hou, Xiaosa Liang, and Zhaoyu Li
*E-mail: yu@physik.rwth-aachen.de, zywang@whu.edu.cn

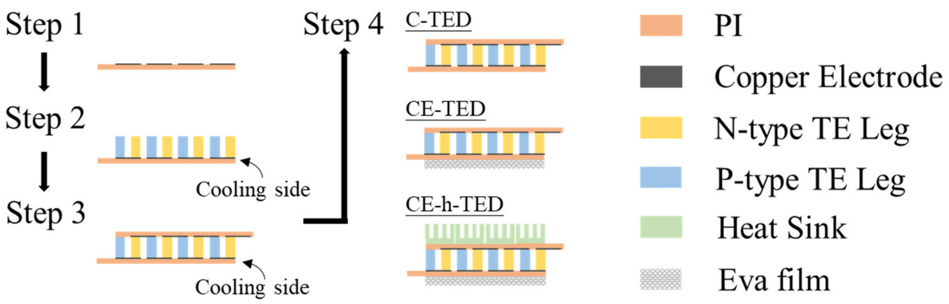

Fig. S1 Fabrication process of the C-TED, CE-TED, and CE-h-TED.

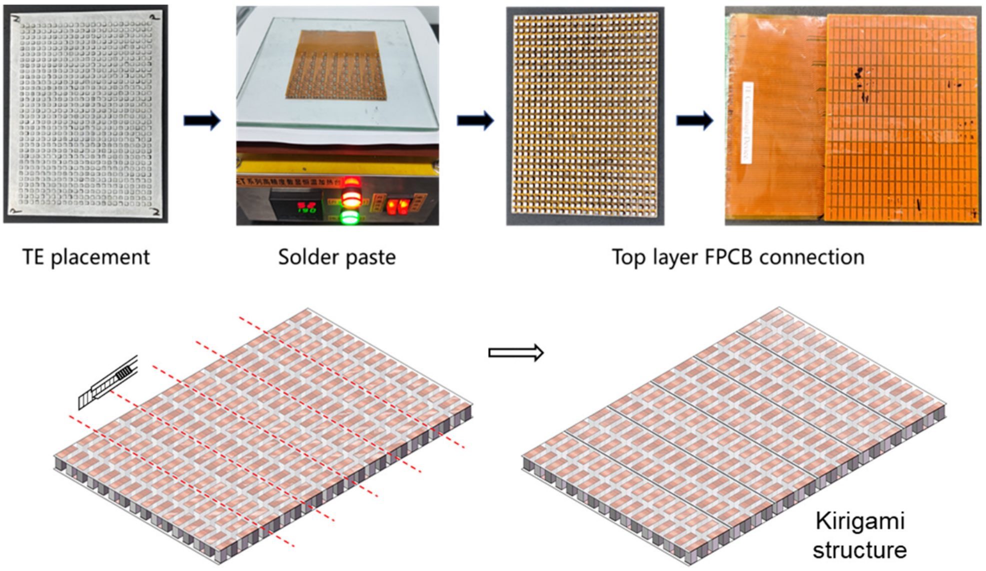

Fig. S2 Fabrication flow chart diagram of the device.

The infrared camouflage devices consisted of a bottom FPCB, a top FPCB, P-type and N-type TE legs, heatsinks, and a top Eva film. The fabrication process included applying solder paste via a stencil, positioning P-type and N-type thermoelectric legs in an aluminum mold, soldering the legs to both FPCBs, and attaching the heatsink and Eva film (Fig. S1). Begin by preparing an auxiliary positioning aluminum plate, 2.0 mm thick, slightly shorter than the thermoelectric legs; Cover one side with polyimide tape to temporarily fix the thermoelectric leg particles; Place the aluminum plate flat on the bench and alternate P-type and N-type particles (see Fig. S2).

Then we apply solder paste uniformly to the FPCB pads using a stencil and position the FPCB over the TE legs on the aluminum plate. In this process, we need to align the pads with the legs. After soldering the TE legs to the bottom electrode, we then need to connect the top electrodes. For soldering the opposite side of the FPCB, the process was identical except that the auxiliary positioning aluminum plate was not required. The final appearance of the fully assembled thermoelectric device array is presented in Fig. S2. To achieve device flexibility, the Kirigami patterning was achieved by first precisely marking cut lines on the PI surface, followed by manual incision along these guides with a surgical scalpel.

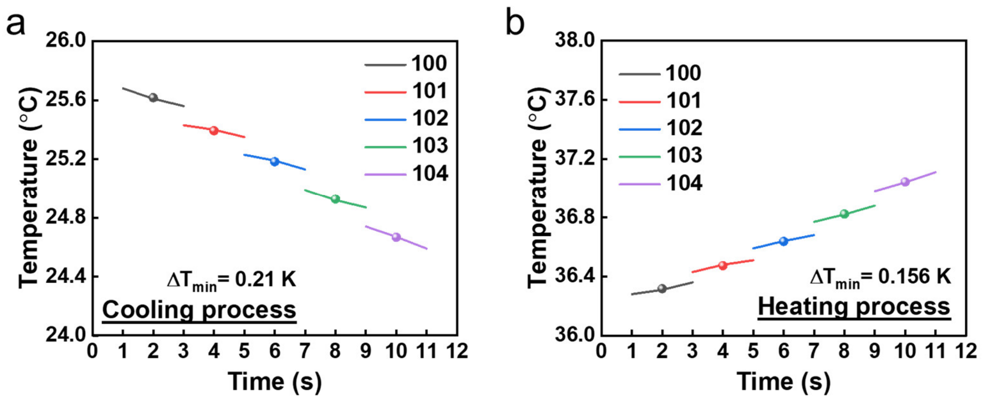

Fig. S3 Minimal resolvable T of Cs-h-TED during the cooling and heating process.

As mentioned in the control interface design of Fig. S3, the relative heating and cooling power levels could be controlled by the number set from -1000 to 1000. Here, we tested the minimal controlling T during the cooling and heating process; the minimal resolvable T were 0.21 and 0.156 K, respectively.

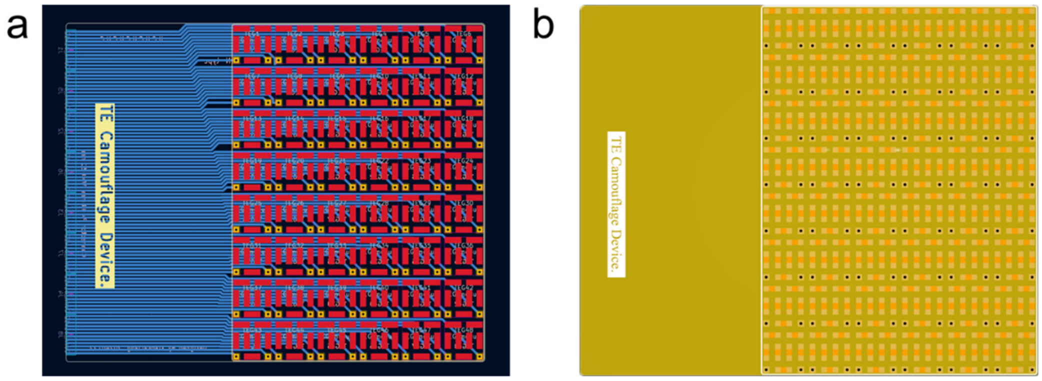

Fig. S4 a. Front and **b.** Back side of the FPCB design of TED.

As the number of thermoelectric devices increases, the overall dimensions of the circuit correspondingly enlarge, with the routing consuming a substantial area of the circuit board, thereby resulting in spatial limitations. Furthermore, excessively narrow trace widths can lead to significant trace resistance, which may cause complications such as overheating and inadequate power delivery. According to the simulations conducted, the rated current required for the thermoelectric devices is 1A, while the peak current is 2A. To ensure that the wire temperature remains within an acceptable range during prolonged operation at the rated current, it is crucial to maintain the cooling effect necessary for infrared camouflage. In circuit design, the temperature differential induced by the heating of printed traces is typically controlled to $\Delta T \leq 10K$ to preserve circuit stability.

In the fabrication process of this system's flexible printed circuit board (FPCB), 1OZ copper foil (approximately 35μm thick) is employed. Under the constraint of maintaining a temperature rise within 10K, the widths of the printed traces are designated as 0.6mm, 0.5mm, and 0.4mm, allowing for maximum currents of 1.6A, 1.35A, and 1.1A, respectively. To ensure the reliable operation of the system and to fulfill the requirements for sustained rated current and transient peak current, while also considering the dimensions and spatial constraints of the FPCB, a trace width of 0.5mm was selected for the design of the FPCB circuitry.

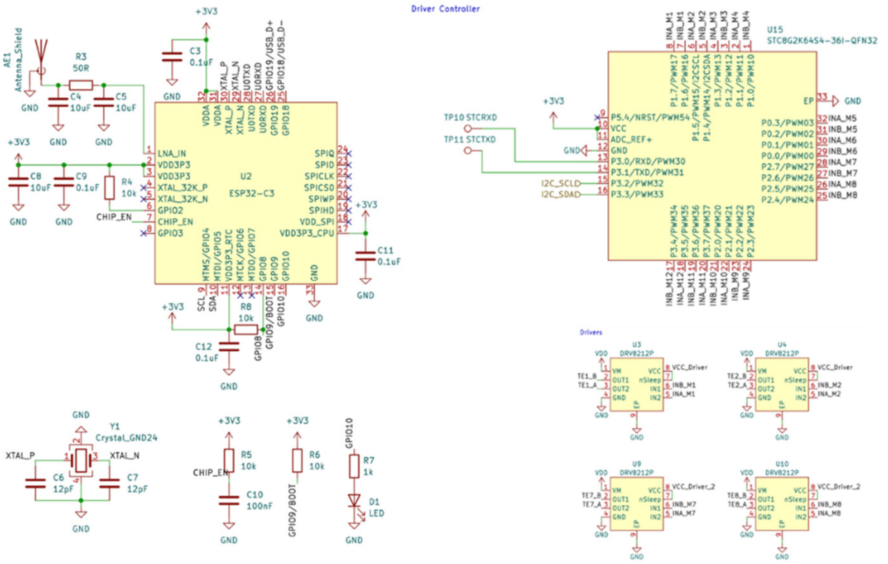

Fig. S5 Design of the controlling circuit. This is a screenshot of KiCad (version 7.0), which contains only KiCad's native user interface.

Figure S5 illustrates the system's power supply and drive control circuits. The main controller circuit, based on the ESP32-C3, is depicted in Fig.S4. To filter out the AC components from the power supply, decoupling capacitors C3, C8, C9, and C11 of varying sizes are connected at the chip's power supply points. The BLE (Bluetooth Low Energy) and WiFi communication antenna circuit is formed by AE1, R3, C4, and C5, with impedance adjusted to 50 Ω for optimal matching. According to the ESP32-C3 datasheet, an external crystal oscillator, comprising Y1, C6, and C7, is required to provide a stable clock source. During power-up, the levels of the GPIO2, CHIP_EN, GPIO9, and GPIO10 pins influence the chip's boot mode; thus, these levels are either pulled high or low to ensure proper system initialization.

In the drive controller circuit, the highly integrated STC8G2K64S4 chip operates reliably using its internal clock circuit, eliminating the need for external components like a crystal oscillator. The P32 and P33 pins serve as the I2C SCL clock and SDA data signals, respectively. Pins P34 to P37, P20 to P27, P00 to P03, and P10 to P17 are designated for PWM signal outputs. The driver chip used is the DRV8212P, capable of directly driving thermoelectric devices. The chip features eight pins: VM (main power supply), VC (logic power supply), GND (common ground), nSleep (sleep mode), IN1 (input 1), IN2 (input 2), OUT1 (output 1), and OUT2 (output 2). The PWM output pins of the drive controller connect sequentially to the IN1 and IN2 pins of the drivers, serving as control inputs. The OUT1 and OUT2 output pairs are connected to the thermoelectric devices, supplying controllable drive currents in both directions and magnitude.

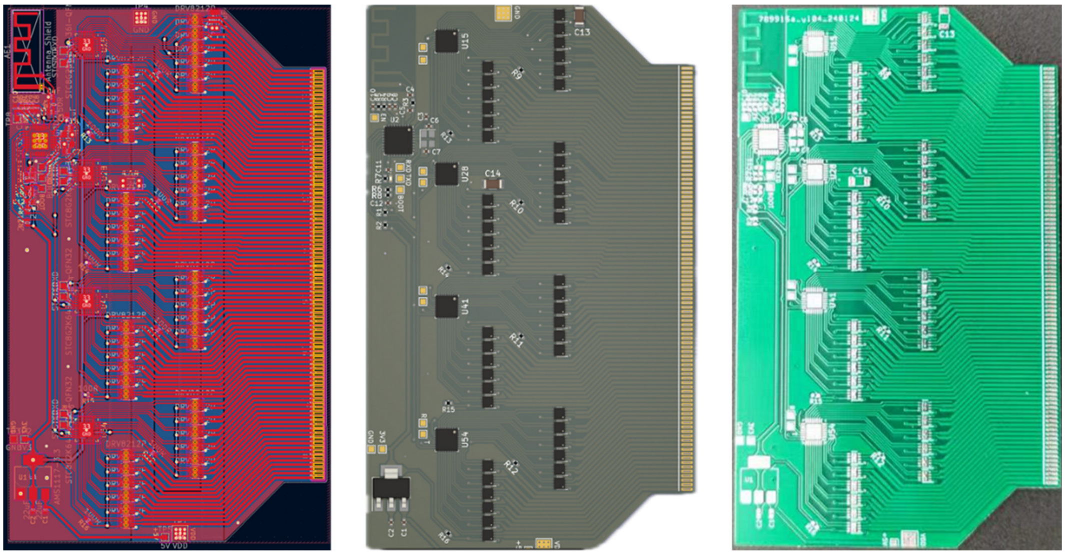

Fig. S6 Design and optical images of the control circuit board.

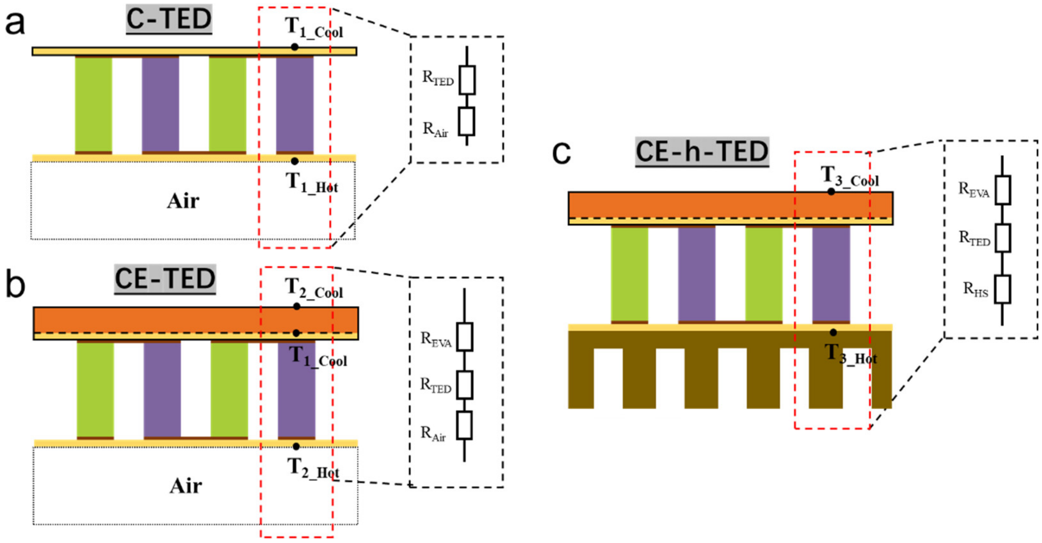

Fig. S7-1 Equivalent longitudinal thermal resistance schematics of single legs for three thermoelectric devices: a. C-TED, b. CE-TED, and c. CE-h-TED.

The thermal image in Fig. 2g was captured at 10 seconds during cooldown, which we deliberately chose this time to ensure fair device comparison, as devices without heat sinks exhibit temperature rebound in later stages due to inadequate heat dissipation, while in the initial phase, C-TED cools faster than heat sink-equipped CE-h-TED. This explains why C-TED appears cooler at 0.5A in Fig. 2g. Based on the heat transfer theory, we analyzed the reasons for the different cooling speeds of the device during the cooling stage below.

Starting from the heat balance equation at the cold side:

$$C_c dTc/dt = -(Q_p - Q_k - Q_{j,c}) \text{ (Eq.1)}$$

Where Cc is the effective thermal capacity at the cold side (including the thermal capacity of the layered structure near the cold side), $Q_p = \alpha ITc$ is the Peltier cooling power (heat absorption), Q_k=G(Th−Tc) is the heat leak from the hot side to the cold side due to thermal conduction (G is the thermal conductance from the cold side to the hot side), and $Q_{j,c}$ is the contribution of Joule heating to the cold side; typically, Joule heating is distributed evenly along the thermoelectric leg, with about half affecting the cold side, i.e., $Q_{j,c} \approx \frac{1}{2} I^2 R$.

At the initial cooling stage (t=0+), $T_c = T_h = T_0$ (ambient temperature), so $Q_k = 0$. Equation 1 simplifies to:

$$C_c dTc/dt = -(\alpha I T_0 - \frac{1}{2} I^2 R) \text{ (Eq.2)}$$

Then the cooling rate can be solved as:

$$\left.\frac{dTc}{dt}\right|_{t=0} = -\frac{\alpha I T_0 - \frac{1}{2} I^2 R}{Cc} \text{ (Eq.3)}$$

The negative sign indicates a temperature drop. If the net cooling power is positive, the initial cooling rate is inversely proportional to *Cc:*

$$\frac{dTc}{dt}\bigg|_{t=0} \propto \frac{1}{Cc} \text{ (Eq.4)}$$

This shows that a smaller cold-side thermal capacity results in a faster initial cooling rate. This is the core reason why C-TED shows the fastest initial cooling speed (Effective cold-side thermal capacity C_c of the three devices: $C_{c,\text{C-TED}}<C_{c,\text{CE-TED}}=C_{c,\text{CE-h-TED}}$). **For effective cold-side thermal capacity C**, as shown in **Fig. S7-1**, adding an EVA porous membrane layer (low thermal conductivity) increases the cold-side thermal capacity $C_{c,\text{CE-TED}}=C_{c,\text{C-TED}}+C_{EVA}>C_{c,\text{C-TED}}$. For CE-h-TED, the hot-side heat sink does not affect the cold side, so $C_{c,\text{CE-h-TED}} = C_{c,\text{CE-TED}}$. Therefore, $\mathbf{C_{c,\text{C-TED}}<C_{c,\text{CE-TED}}=C_{c,\text{CE-h-TED}}}$.

For the subsequent process (t>0), the temperature change follows an exponential decay with time constant τ=C/G (C is the total system thermal capacity, approximated as cold-side thermal capacity C_c; G is the total internal thermal conductance). Assuming a small temperature difference, solving the heat equation yields an exponential form:

$$Tc(t)=T_0-\Delta T_{\max}(1-e^{-t/\tau}) \text{ (Eq.5)}$$

The general cooling rate expression is:

$$\frac{dTc}{dt} = -\frac{\Delta T_{max}}{\tau}e^{-t/\tau} \text{ (Eq.6)}$$

Where ΔT_{max} is the maximum possible temperature difference. Thus,

$$\frac{dTc}{dt}\bigg|_{t=0} \propto \frac{G}{C_c} \text{ (Eq.7)}$$

For internal thermal conductance G, the EVA porous membrane layer has low thermal conductivity, increasing the thermal resistance from the cold side to the thermoelectric leg, thus reducing thermal conductance: $G_{\text{CE-TED}}<G_{\text{C-TED}}$. For CE-h-TED, the hot-side heat sink only affects the thermal resistance from the hot side to the environment, not the internal thermal conductance (from cold side to hot side), so $G_{\text{CE-h-TED}}=G_{\text{CE-TED}}$. Thus, $G_{\text{C-TED}}> G_{\text{CE-TED}}= G_{\text{CE-h-TED}}$.

Then, coming back to Eq.7, we can conclude that device C-TED has the fastest cooling rate in the initial stage because it has the smallest cold-side thermal capacity and the highest thermal conductance.

The lowest temperature that can be achieved by Peliter cooling ($T_{c,\min}$) of a thermoelectric cooler (TEC) is determined by the steady-state heat balance. When the heat absorption at the cold junction (Peltier effect) balances with the heat dissipation capability at the hot side, heat leakage, and Joule heating, $dT_c/dt=0$, and the cold-side temperature stops decreasing. The steady-state equation is:

$$\alpha IT_c-\frac{1}{2}I^2R=G(T_h-T_c) \text{ (Eq.8)}$$

Then, the expression for minimum temperature (neglecting environmental heat exchange) is:

$$\Delta T_{\max}=T_h-T_{c,\min}=\frac{\alpha ITc-\frac{1}{2}I^2R}{G} \text{ (Eq.9)}$$

From equation 9, we can conclude that $T_{c,\min}$ is inversely related to G (**smaller G is better for deep cooling**), albeit at the cost of slower cooling speed, and reducing T_h is the key to breaking the limit of $T_{c,\min}$. For CE-h-TED, it has a smaller G value when compared with C-TED (previously explained in question 8 with the result of $G_{C\text{-}TED} > G_{CE\text{-}TED} = G_{CE\text{-}h\text{-}TED}$) and smaller T_h when compared with CE-TED (Metal heat sink significantly lowers T_h), resulting in the smallest $T_{c,\min}$ value within these three devices when reaching steady-state.

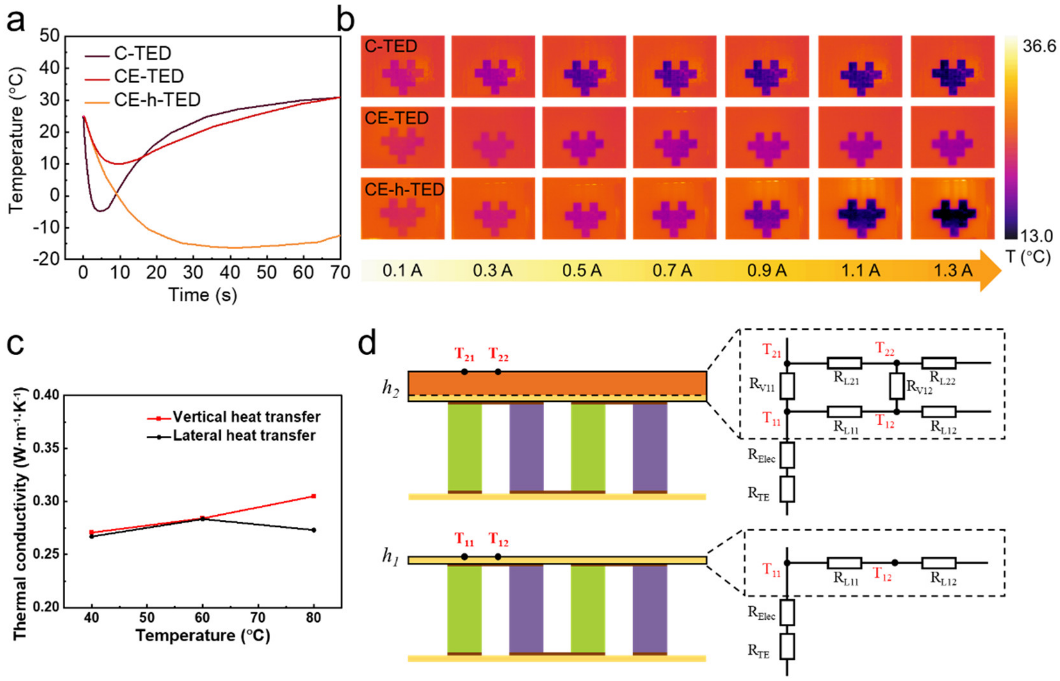

Fig. S7-2 a. COMSOL-simulated temperature evolution on thermoelectric surfaces of three devices (C-TED, CE-TED, CE-h-TED) under the driving current of 1.3 A; **b.** Infrared images of device surfaces under varying driving currents for C-TED, CE-TED, and CE-h-TED as reported in the original text. **c.** Vertical and lateral thermal conductivity of the EVA sponge layer. **d.** Equivalent thermal resistance model with and without Eva layer

The COMSOL simulations in Fig. S7-2a also verify that the C-TED is the coolest at 0.5 A and CE-h-TED is the coolest at 1.3 A in Figure 7-2b. During the initial cooling phase (especially within the first 10 seconds), the temperature drop-rate of the heatsink-equipped CE-h-TED is significantly slower than that of the heatsink-free devices (C-TED and CE-TED). This delayed transient response explains why C-TED exhibits the lowest temperature at the 0.5 A measurement point in S7-2b.

We measured the thermal conductivity of the EVA film (Fig. S7-2c). Results show that the in-plane transverse thermal conductivities at 40°C, 60°C, and 80°C are 0.267, 0.284, and 0.273 W/(m·K), respectively, while the longitudinal values are 0.271, 0.284, and 0.305 W/(m·K). The longitudinal conductivity is slightly higher than the transverse, though the difference is insignificant at lower temperatures. By comparing the transverse/longitudinal heat transfer characteristics of the device before and after EVA film integration (Fig. S7-2d), we further elucidate the mechanism by which EVA blurs bright-dark boundaries in displays. According to the heat transfer equation:

$$\Delta T = Q \cdot Rt \quad (1)$$

where ΔT denotes the temperature difference between two surface points, Q is the heat flux, and *Rt* is the thermal resistance. The enhanced transverse uniformity after adding a 2 mm EVA film is explained as follows:

According to the thermal resistance formula:

$$R = h/\lambda \cdot A \quad (2)$$

where *h*, *λ*, and *A* are thickness, thermal conductivity, and heat transfer area, respectively. Indicating that under identical A, the EVA sponge layer (2 mm) exhibits substantially higher thermal resistance R than the PI layer (0.1 mm), thereby impeding longitudinal heat transfer. Equation (1) yields $T_{21}<T_{11}$, explaining the observed contraction of the temperature range after adding the Eva film.

The bright-dark boundary contrast, governed by T_{21}-T_{22} and T_{11}-T_{12}, is reduced because EVA increases the total thickness. This creates additional transverse conduction paths, reducing transverse heat flux Q_{21} when compared with Q_{11} in the device without EVA film. Consequently, Equation (1) implies that decreased Q_{21} lowers T_{21}-T_{22}, leading to a more uniform temperature distribution.

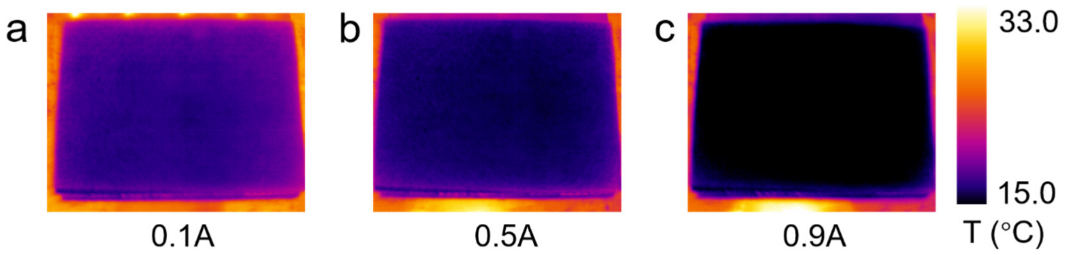

Fig. S8 Cooling display performance of the CE-h-TED under high current conditions.

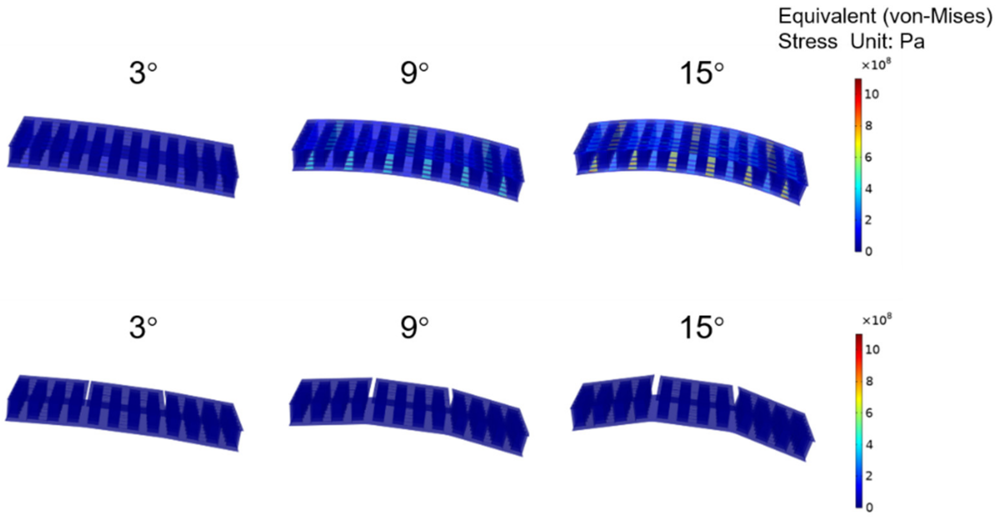

Fig. S9 Stress distribution for devices with/without Kirigami structure

Device flexibility enhancement originates from stress release via this kirigami structure, as validated by COMSOL simulations comparing devices with/without Kirigami (**Fig. S9**). At 15° bending, peak stresses on the PI surface were 297 MPa (uncut) versus 60.4 MPa Kirigami-structured), demonstrating a 79.7% reduction and confirming the mechanism’s efficacy.

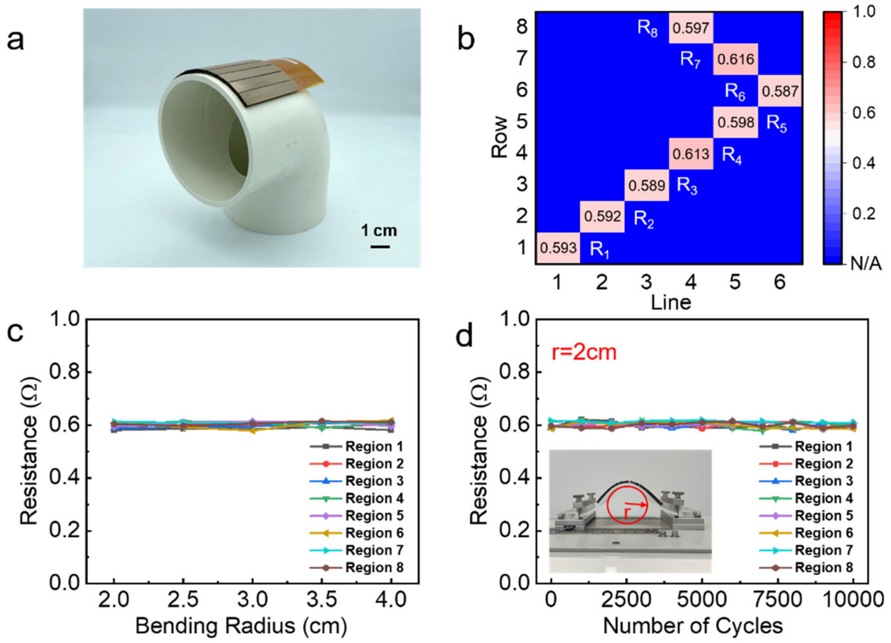

Fig. S10 Flexibility and stability testing of the IR-TED. a. Photograph of C-TED attached to a curved surface. **b.** The internal resistance of eight regions randomly chosen from each row of the TED panel. **c.** The resistance variation of the eight-pixel region when the TED panel was bent in different bending radii (R is from 2 cm to 4 cm with 0.5 cm in between). **d.** The resistance variation of eight-pixel regions for 10000 bending cycles with a bending radius of 2 cm.

As shown in **Figure S10a**, the device could bend and conform well to a cylindrical surface with a radius of 5 cm. To verify the flexibility and stability of the device during long-term bending, we selected a single thermoelectric unit from each row and tested its initial internal resistance. The internal resistance of the eight randomly selected units, from the bottom to top, accorded to the number of rows (R_1 to R_8), ranged from 0.59 Ω to 0.62 Ω, with a maximum fluctuation not exceeding 0.03 Ω, indicating both the reliability of the device fabrication and the acceptable design of the copper trace in FPCB.

In Fig. S10c, we placed the device on a silicone sheet and stretched it to cover cylindrical surfaces with varying curvature radii, decreasing from 4 cm to 2 cm in 0.5 cm increments. As shown in the bending test results for the eight regions in Figure 4c, the maximum internal resistance change did not exceed 3.93%, demonstrating excellent bendability. The cycling bending tests in Figure 4d show that after 10,000 bending cycles, the maximum internal resistance change in the eight regions did not exceed 5.79%, further validating the device's stability during long-term bending use.

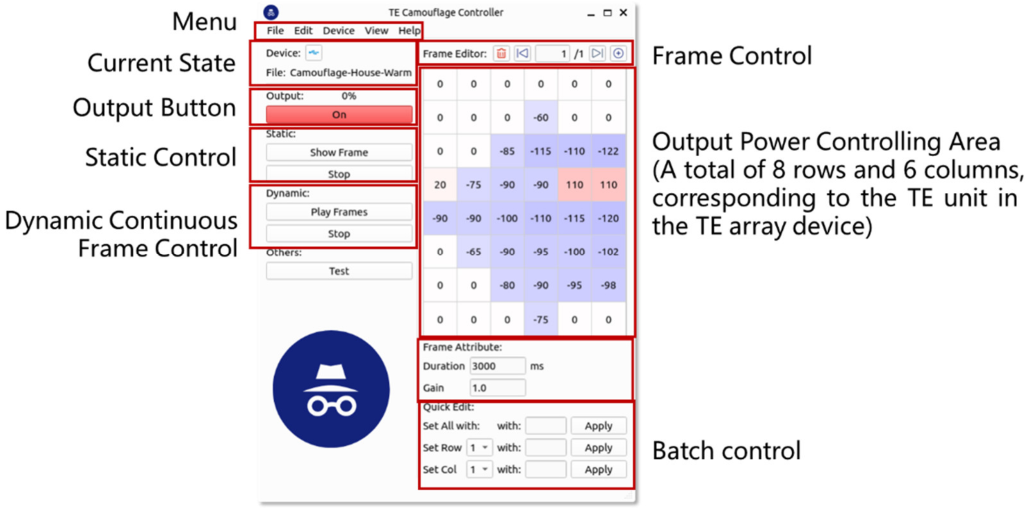

Fig. S11 Control interface design. This is a screenshot of the author's self-developed host-computer software platform without third-party assets embedded.

End-users predominantly interact with hardware devices through applications. In pursuit of superior human-computer interaction and to augment system usability, we have employed the Qt open-source, cross-platform application development framework to engineer an infrared camouflage control system application.

The inherent cross-platform capability of Qt offers users seamless deployment across a variety of computing environments, including but not limited to Windows, macOS, and Linux systems, thereby facilitating multi-device accessibility. For development teams, the Qt framework comes equipped with Qt Designer, a tool designed for graphical user interface creation and editing. This tool supports a drag-and-drop interface for element placement and layout modification, thereby streamlining the development process and enhancing productivity.

Illustrated in **Figure S11** is the user interface of the infrared camouflage control system developed using Qt. After establishing a connection between the user's device and the flexible thermoelectric camouflage apparatus via BLE or a serial cable, the interface allows for the regulation of the thermoelectric camouflage system's output. The interface features a menu bar at the top, offering options for file management, data frame editing, device control, view settings, and access to help documentation. The primary interface area is segmented; the left portion comprises quick access information and buttons to promptly activate device functionalities. Conversely, the right section graphically represents the relative heating and cooling power levels required for the manipulation of the infrared image, with a range from -1000 to 1000, signifying the lowest cooling power to the highest heating power, respectively. Upon entering or outputting power values within the interface's table, these values are instantaneously translated into corresponding colors based on a predefined color map. After initiating the control sequence, the interface transmits the control parameters to the hardware MCU through either a wireless or wired medium, enabling the hardware to adjust its operational parameters in accordance with the heating or cooling power settings defined

by the user.

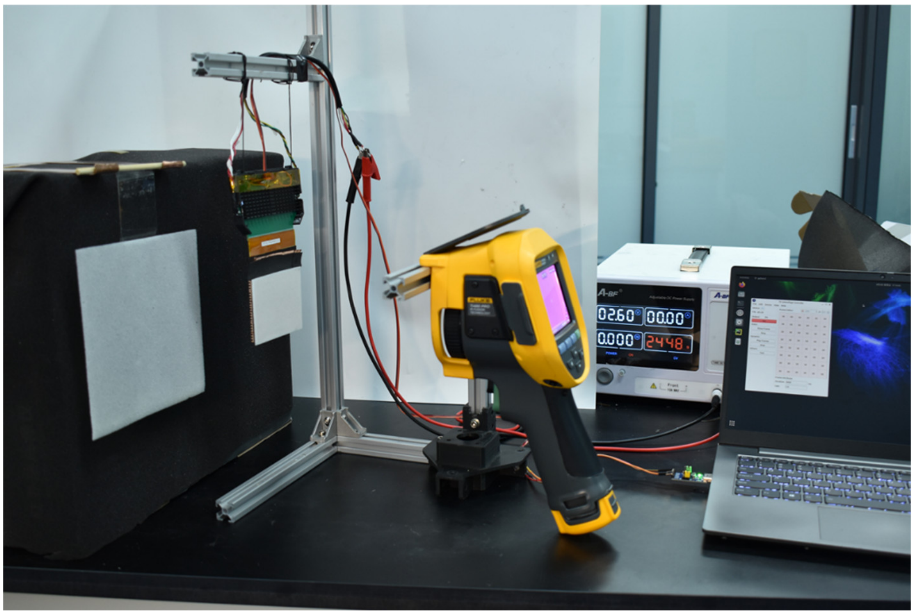

Fig. S12 Testing setup for the active thermal concealment

The testing setup is shown in Figure S12, which consists of an IR camera, testing device, movable background, controlling panel (computer), and power source. The moving car or triangular model demonstrated in the manuscript is stuck on a box and then pushed into the view of the IR camera.

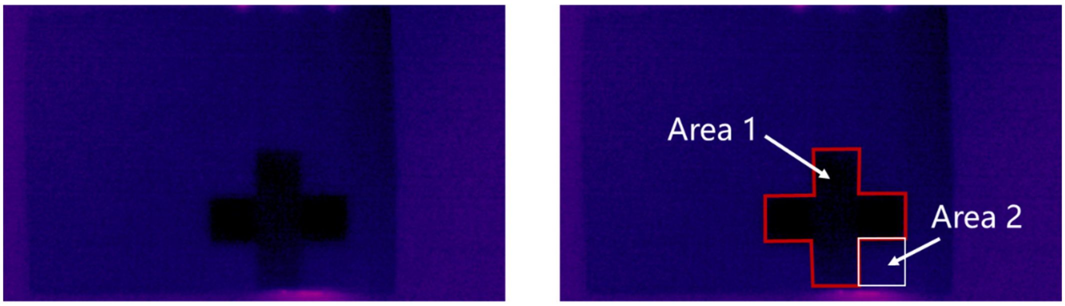

Fig. S13 Simultaneously achieve IR concealment and IR camouflage through independent area T control.

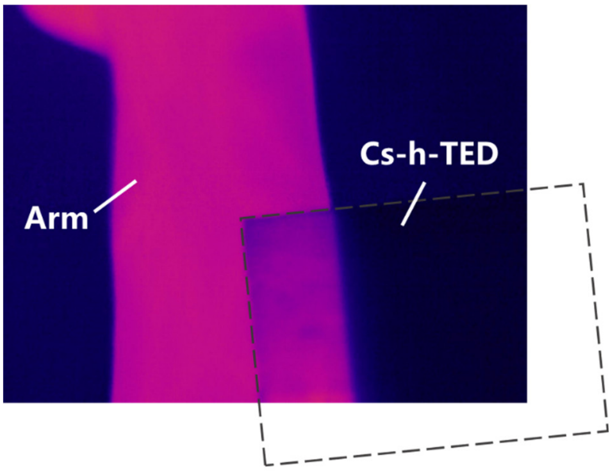

Fig. S14 Self-hiding ability when CE-h-TED was placed on our human arm.

The CE-h-TED was also capable of self-hiding when carried around. As shown in **Fig. S14**, when placed in front of our arm, the device itself could be hidden under the IR camera.

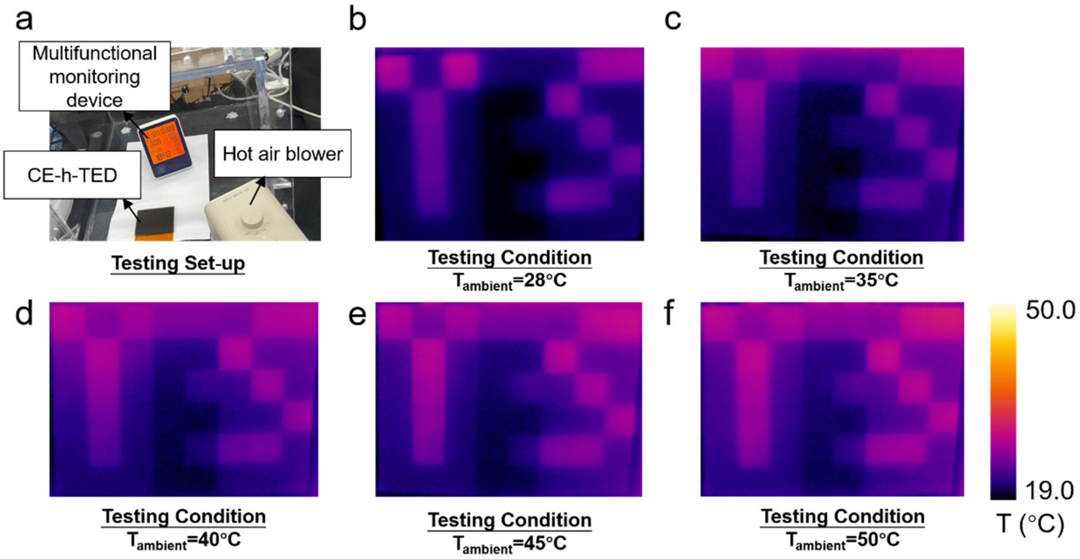

Fig. S15 Thermal information display under varying ambient temperatures. a. Experimental setup; **b-f.** Functional demonstration of the "YES" character display across progressively increasing thermal environments.

For dynamic background testing, we introduced hot air into the chamber to elevate the ambient T (T_{amb}) from 28°C to 50°C (**Fig. S15a**). Results in Fig. S15b-f demonstrate that as T_{amb} increased, the cooled "E" in our "YES" demo actually became more visible from Fig. R15b to c (hotter background means better cool-spot contrast!). As the T_{amb} kept going up, the whole image shown on the display became hotter, with the heated “YS” turning brighter and the cooled letter "E" becoming brighter.

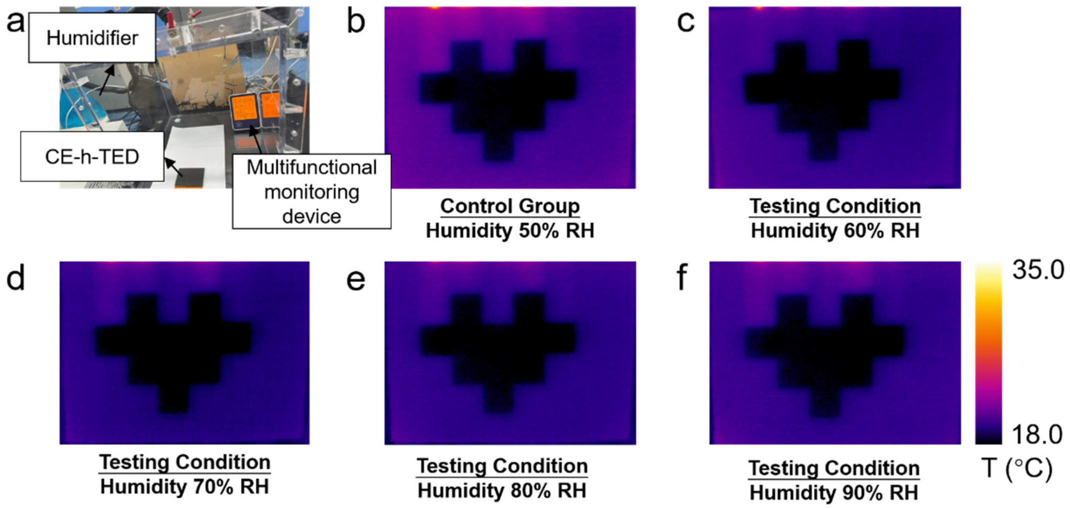

Fig. S16 Imaging performance of CE-h-TED under humidity gradients. a. Humidity test chamber setup; **b-f.** Functional demonstration across varying relative humidity (RH) levels with sustained thermal display fidelity.

For humidity testing (**Fig. S16a**), a humidifier increases chamber humidity levels from 50% to 90% RH. The device maintained full functionality across this range (Fig. S16b-f). Notably, no electrical shorts occurred even at 90% RH, where visible condensation formed on chamber walls, with thermal images remaining clear.

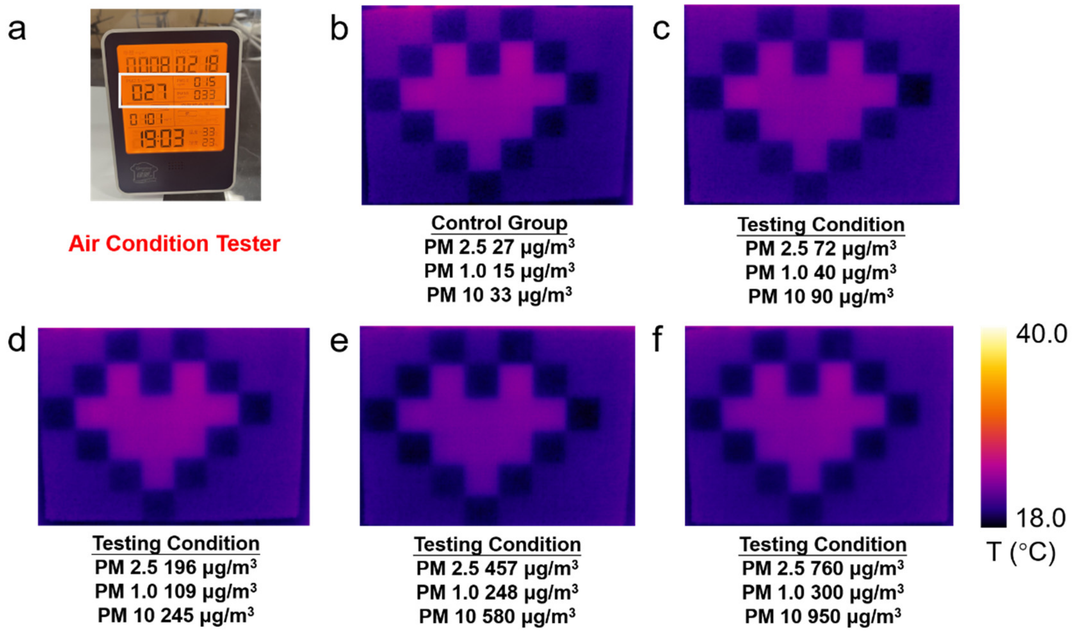

Fig. S17 Imaging Performance of CE-h-TED in varied dust concentrations. **a.** Air condition tester in ambient air; **b-f.** Functional demonstration under increasing dust levels (PM2.5, PM1.0 and PM10) within a half-sealed chamber.

For dust testing, the device was placed in a half-sealed acrylic chamber (38 cm × 38 cm × 38 cm, L × W × H) with an IR camera port. Standardized electronic-grade artificial dust (sourced from Zhongwei Instruments) was blown inside the chamber incrementally. Results (**Fig. S17**) confirm normal thermal operation at all dust concentrations, though image clarity gradually decreased at higher concentrations. We analyzed that it might be due to IR scattering.

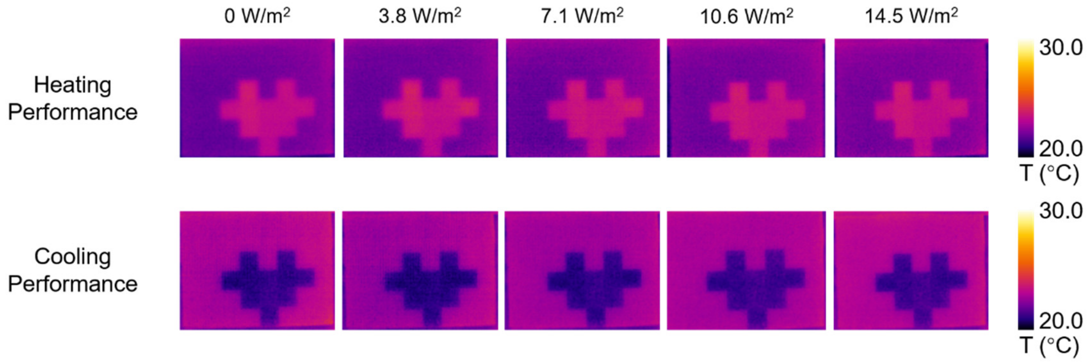

Fig. S18 Heating/Cooling performance of the device surface under different light radiation.

For sunlight changes, we have bought an adjustable sun simulator lamp that covers all light spectrum and turned it on from total darkness up to bright, with the brightness ranging from 0 to 14.5 W/m^2. As shown in **Fig. S18**, the heart-shaped image, both in cooling and heating performance, could be clearly demonstrated on the panel. Though honestly, super bright light (high radiation) does heat the whole testing area (more experiment details are shown in ambient temperature effects below).

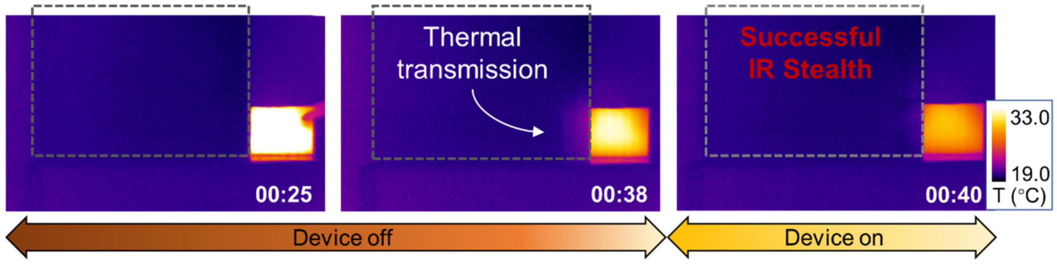

Fig. S19 IR Concealment performance for CE-TED.

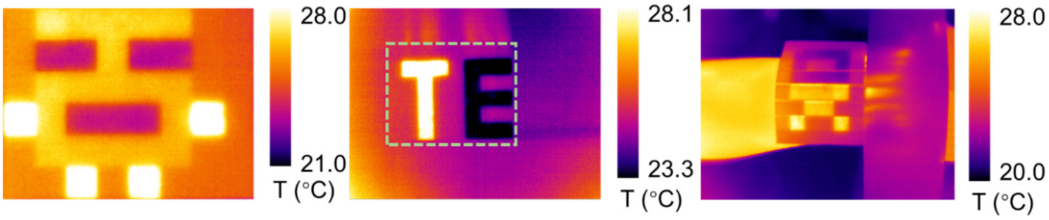

Fig. S20 Images and message display on the IR-TED based on both low and high temperatures.

Other applications, including the demonstration of the cartoon pattern, a message of the capital letter "TE" in the plane state, and a message of the capital letter "OK" in the bent state, are shown in **Fig. S20**.

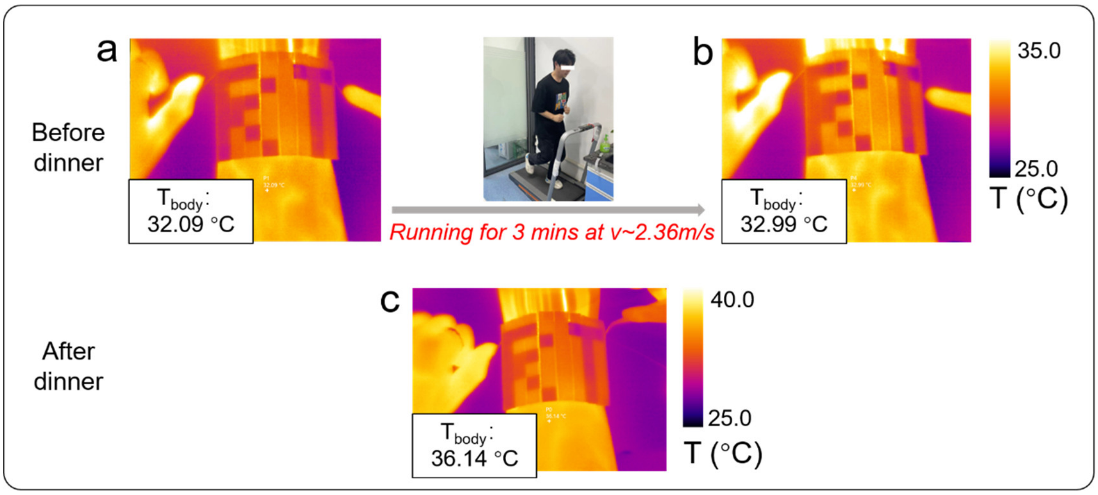

Fig. S21 On-Body thermal display under different body temperatures. Image of "RT" letter (Running Test for short) shown on the bending display (CE-TED) under (a) Pre-exercise state; (b) Post-3min fast treadmill running state; and (c) Postprandial metabolic elevation state.

In **Fig. S21**, the volunteer was wearing the CE-TED on his wrist, and the device performance of the letter "RT" ("Running Test" for short) was tested under the pre-exercise state and post-exercise state. After 3 min of fast treadmill running, the wrist skin temperature increased slightly (+0.9°C vs. pre-exercise state in Fig. R14a) with no distinct degradation in "RT" character recognition. Additionally, after dinner, a significant wrist temperature increase of 4.05°C (reaching 36.1°C) was observed in Fig. 14c, yet the device maintained full functionality, achieving complete recognition of the test letters. These findings indicate that within natural thermophysiological ranges (fluctuations <5°C), CE-TED performance is not significantly affected by epidermal temperature variations, ensuring reliable operation during human activities.

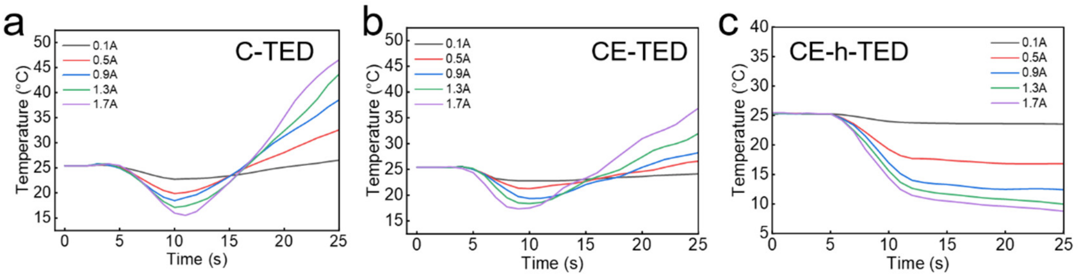

Fig. S22 Surface temperature of C-TED, CE-TED, CE-h-TED under identical driving conditions (0.1A-1.7 A, 0.4A in between, 25.4°C ambient)

We conducted additional thermocouple measurements (K-type, 1 mm diameter, ±0.5°C accuracy) on the surfaces of all three devices (**C-TED, CE-TED, CE-h-TED**) under identical driving conditions (0.1A-1.7 A, 0.4A in between, 25.4°C ambient). As shown in **Fig. S22**, the tested cooling trend for all three devices aligns well with the data we tested by the IR camera.

Table S1. Power consumption for the TED-based system.

	1	2	3	4	5	6	7	8	9	10
Applied Voltage (V)	3	3	3	3	3	3	3	3	3	3
Applied Current for Whole System (A)	0.38	0.59	0.78	0.98	1.18	1.36	1.57	1.77	1.95	2.14
Power Consumption (W)	1.14	1.77	2.34	2.94	3.54	4.08	4.71	5.31	5.85	6.42

Table S2. The estimated power consumption and its battery life under different operating modes.

**Operation Mode**	**System Current (A)**	**Power (W)**	**Energy/Cycle (J)**	**Endurance**
**Active Mode (~1.9A for single pixel)**	2.14	6.42	~38.52	~1.15 hours
**Baseline Concealment (~0.1A for single pixel)**	0.38	1.14	~3.42	~6.49 hours

The system operates within a **power range of 1.08–5.64 W** (0.36–1.88 A @ 3V DC), with complete energy profiles detailed in **Supplementary Table S1**. While peak power states (e.g., 6.42 W at 2.14A/3V) present challenges for continuous battery operation, the following strategies ensure viable portable and long-duration deployment:

1) Active high-power mode: Active camouflage requires only **6 s per operation** at 1.9 A (Fig. 3a), consuming **38.52 J/cycle**. This enables **>690 cycles (~1.15 h)** on a single 18650 cell (3.7V, 2000mAh, 7.4 Wh)- compact enough (Φ18×65 mm) for portable integration.

2) Low-power Mode: Maintaining baseline concealment at **1.14 W** (0.38 A) extends operational endurance by ~5 times compared to sustained high-power modes.

Table S3. Weight for C-TED, CE-TED, and CE-h-TED.

	C-TED	CE-TED	CE-h-TED
Weight (g)	32.24	32.48	93.20
